# Supplementary material for: Mechanisms governing the pioneering and redistribution capabilities of the non-classical pioneer PU.1
Source: Nat Commun. 2020 Jan 21;11:402. doi: 10.1038/s41467-019-13960-2 (PMC6972792; doi:10.1038/s41467-019-13960-2)
Supplement: Supplementary file 7 — Source data [file 41467_2019_13960_MOESM7_ESM.zip › Source_Data/FigureS2/FigureS2B_MotifScanOutput/KmeansCluster1/homerResults/motif20.similar.html]

motif20

## Information for motif20

C
T
A
G
G
A
T
C
G
T
C
A
G
C
A
T
G
A
T
C
A
G
T
C
C
G
T
A
T
C
G
A
C
T
G
A
A
G
C
T
C
G
A
T
T
A
C
G
C
T
A
G
  
Reverse Opposite:  

A
G
T
C
A
G
T
C
C
G
T
A
T
C
G
A
A
G
C
T
A
G
C
T
A
C
G
T
C
T
A
G
C
T
A
G
C
G
T
A
A
C
G
T
C
T
A
G
G
A
T
C
  

|  |  |
| --- | --- |
| p-value: | 1e-40 |
| log p-value: | -9.398e+01 |
| Information Content per bp: | 1.777 |
| Number of Target Sequences with motif | 72.0 |
| Percentage of Target Sequences with motif | 1.41% |
| Number of Background Sequences with motif | 37.6 |
| Percentage of Background Sequences with motif | 0.09% |
| Average Position of motif in Targets | 102.6 +/- 25.4bp |
| Average Position of motif in Background | 113.7 +/- 68.9bp |
| Strand Bias (log2 ratio + to - strand density) | 0.1 |
| Multiplicity (# of sites on avg that occur together) | 1.00 |
| Motif File: | file (matrix) reverse opposite |

### Similar de novo motifs found

|  |  |  |  |  |  |  |  |
| --- | --- | --- | --- | --- | --- | --- | --- |
| Rank | Match Score | Redundant Motif | P-value | log P-value | % of Targets | % of Background | Motif file |
| 1 | 0.912 | A G T C C G T A G C A T G A T C G T A C G T C A C T G A T C G A A G C T C G A T C A T G | 1e-34 | -79.553167 | 1.87% | 0.29% | motif file (matrix) |
| 2 | 0.894 | C T G A A G C T A G C T C G A T C T A G C T A G C G T A A C G T C T A G A G T C | 1e-31 | -73.077848 | 1.85% | 0.32% | motif file (matrix) |
| 3 | 0.802 | C G T A C A G T A G T C G T A C G C T A C T G A C G T A G A C T C G A T C T A G C T A G C T G A | 1e-29 | -66.795097 | 1.24% | 0.13% | motif file (matrix) |
| 4 | 0.845 | A C T G A G T C C G T A A C G T A G T C A G T C C G T A C T G A C G T A | 1e-15 | -35.870113 | 2.02% | 0.73% | motif file (matrix) |
